# Dengue illness index—A tool to characterize the subjective dengue illness experience

**DOI:** 10.1371/journal.pntd.0006593

**Published:** 2018-10-04

**Authors:** Stephen J. Thomas, Liane Agulto, Kim Hendrickx, Martin Erpicum, Kay M. Tomashek, M. Cristina Cassetti, Catherine Laughlin, Alexander Precioso, Alexander C. Schmidt, Federico Narvaez, João Bosco Siqueira, Hasitha Tissera, Robert Edelman

**Affiliations:** 1 Division of Infectious Diseases, State University of New York, Upstate Medical University, Syracuse, NY, United States of America; 2 Division of Microbiology and Infectious Diseases, National Institute of Allergy and Infectious Diseases, National Institutes of Health, Rockville, MD, United States of America; 3 FWO Postdoctoral Fellow of the Life Sciences & Society Lab, Center for Sociological Research, KU Leuven, Flanders, Belgium; 4 SPIRAL Research Center, Département de Science Politique, Université de Liège, Liège, Belgium; 5 Division of Clinical Trials and Pharmacovigilance, Butantan Institute, São Paulo, Brazil; 6 Pediatrics Department of the Medical School of University of São Paulo, São Paulo, Brazil; 7 GSK Vaccines, Rockville, MD, United States of America; 8 Infectious Diseases Unit, National Pediatric Reference Hospital, Hospital Infantil Manuel de Jesús Rivera, Managua, Nicaragua; 9 Federal University of Goias, Brasilia, Brazil; 10 National Dengue Control Unit, Ministry of Health, Colombo, Sri Lanka; 11 Center for Vaccine Development, University of Maryland School of Medicine, Baltimore, MD, United States of America; University of California San Francisco, UNITED STATES

## Abstract

Dengue virus infections are a major cause of febrile illness that significantly affects individual and societal productivity and drives up health care costs principally in the developing world. Two dengue vaccine candidates are in advanced clinical efficacy trials in Latin America and Asia, and another has been licensed in more than fifteen countries but its uptake has been limited. Despite these advances, standardized metrics for comparability of protective efficacy between dengue vaccines remain poorly defined. The Dengue Illness Index (DII) is a tool that we developed thru refinement of previous similar iterations in an attempt to improve and standardize the measurement of vaccine and drug efficacy in reducing moderate dengue illness. The tool is designed to capture an individual’s overall disease experience based on how the totality of their symptoms impacts their general wellness and daily functionality. We applied the DII to a diary card, the Dengue Illness Card (DIC), which was examined and further developed by a working group. The card was then refined with feedback garnered from a Delphi methodology-based query that addressed the adequacy and applicability of the tool in clinical dengue research. There was overall agreement that the tool would generate useful data and provide an alternative perspective to the assessment of drug or vaccine candidates, which in the case of vaccines, are assessed by their reduction in any virologically confirmed dengue of any severity with a focus on the more severe. The DIC needs to be evaluated in the field in the context of vaccine or drug trials, prospective cohort studies, or during experimental human infection studies. Here, we present the final DIC resulting from the Delphi process and offer its further development or use to the dengue research community.

## Author summary

The Dengue Illness Index (DII) is a tool that was developed to improve and standardize the measurement of vaccine and drug efficacy in reducing moderate dengue illness by capturing the overall subjective disease experience of an individual based on how the totality of their symptoms impacts their wellness and daily functionality. The DII was applied to a diary card, the Dengue Illness Card (DIC), which was examined and further developed by a working group. The resulting DIC was then refined with feedback garnered from a Delphi methodology-based query that addressed the adequacy and applicability of the card in dengue research. Here, we report on the Delphi results and present the final DIC.

## Introduction

Dengue is an arboviral illness caused by infection with any of the four dengue virus (DENV) types named DENV-1, -2, -3, or -4. The DENVs are flaviviruses in the same family as Japanese encephalitis, Yellow fever, West Nile, and Zika viruses, and a number of other encephalitic viruses. Although case fatality rates and overall global mortality may be lower than other vector borne diseases such as malaria, the public health impact of dengue transmission and disease is considerable. There is an estimated 396 million DENV infections each year globally of which approximately 96 million are clinically apparent.[1] Dengue significantly affects both financial and other measures of productivity such as disability-adjusted life years and causes significant morbidity and drives up health care costs principally in the developing world.(DALYS).[2, 3] Dengue is a major cause of febrile illness in returning travelers, especially from Southeast Asia, Latin America and the Caribbean.[4] Military deployed to dengue endemic regions are also at risk of infection and dengue has been identified as an important cause of febrile illnesses and disability during numerous operations.[5, 6]

Following a DENV infection an individual may experience a broad range of clinical outcomes. These include a clinically unapparent infection, a mild febrile illness, which may not motivate health care-seeking behavior, or a spectrum of clinical phenotypes from mild to severe prompting engagement with a medical provider.[7] Mild to moderate disease may manifest as fever plus a number of symptoms to include muscle and bone pain, headache and eye pain, nausea, fatigue, and other non-specific symptoms. Uncomplicated dengue may have bleeding manifestations but without significant end organ damage or clinically significant intravascular volume loss or coagulopathy. Bleeding gums, epistaxis, heavy menses, bruising and bleeding from sites of needle insertion may occur but by themselves may not be associated with a poor clinical outcome. There also may be evidence of slight plasma leakage into the abdominal, pericardial, and pleural space but, again, such capillary leakage may not be clinically significant. By contrast, severe dengue most often occurs as the result of significant plasma leakage and coagulopathy resulting in hypotension or shock. The resulting decreased perfusion of end organs such as the liver, kidneys, heart and/or gastrointestinal tract may result in ischemia and subsequent organ dysfunction manifested as end organ failure, and potentially death.

No specific DENV antiviral drug or immunotherapeutic currently exits. Attempts to repurpose unlicensed compounds or already licensed drugs with a different indication have failed to produce a therapeutic that consistently reduces dengue disease severity or improve clinical outcome.[8–11] } A dengue vaccine has been licensed in more than fifteen dengue endemic countries. The vaccine’s performance against preventing severe and hospitalized dengue was significantly superior to its ability to prevent mild or moderate dengue in seropositive vaccine recipients. An observed increase in the relative risk of severe or hospitalized dengue in the dengue naïve and youngest age group during year three of the Phase III trial raised concerns about the potential for dengue vaccines to increase the occurrence of symptomatic disease.[12] Initial concerns are now supported by long term safety data indicating variance in vaccine performance between individuals who are seropositive prior to vaccination compared to those who are seronegative; the latter experience a higher risk of severe or hospitalized dengue following natural infection. The World Health Organization Strategic Advisory Group of Experts recently modified their previous recommendations regarding the vaccine and have advised only vaccinating seropositive individuals. These new data and a primary vaccine series of three doses administered over a year justifies the development and testing of better performing second-generation vaccines with superior target product profiles.[12–14] There are currently two dengue vaccine candidates in advanced clinical efficacy trials in Latin America and Asia and a number of other candidates in early clinical development.[15, 16]. In the current paper, the Dengue Illness Index (DII) tool was adopted and re-engineered from previous versions (Dengue Severity Index and Reactogenicity Index).[17, 18] Our purpose was to improve and standardize the measurement of vaccine and drug efficacy in reducing moderate dengue illness, which represents the overwhelming share of dengue burden in the world.[3, 19]

### Capturing and characterizing dengue cases in clinical trials

Interventional efficacy trials need to prospectively define criteria for what constitutes a “case” of dengue. Defining what will and will not be considered a “case” is not a simple task. It encompasses a number of considerations and nuances. The definition must be objective and easily measurable. It must not require prohibitively expensive or complex technology to complete the measurements. The definition must be applicable across countries and cultures where health care seeking behavior and the standard approach to medical care delivery may vary, even in the context of a highly regulated and controlled clinical trial. Ideally, the case definition should represent the public health burden the intervention is seeking to relieve. “Cases” should also occur with sufficient frequency within a proposed study population, such that the conduct of an efficacy trial is financially and logistically feasible within a reasonable timeframe.

For dengue vaccine trials, developers/sponsors have used some variation of fever plus a positive reverse transcriptase-polymerase chain reaction (RT-PCR) and/or an anti-dengue non-structural protein 1 (NS1) serological assay as the basis for identifying a “case.” The prevention of fever plus RT-PCR or anti-NS1 positivity in the vaccine group compared to the control/placebo group defines the primary efficacy endpoint. Secondary endpoints in these trials typically include severe dengue phenotypes or hospitalized disease (ClinicalTrials.gov Identifier: NCT02747927, NCT02406729). We hypothesize there is a spectrum of clinically relevant dengue falling between the endpoints of more mild dengue (most consistent with the definition of a “case” (via supra) and more severe dengue cases (defined in trials as “severe” or hospitalized cases). By failing to measure clinical disease events falling between these endpoints, vaccine or drug efficacy may be underestimated. Specifically, a vaccine may not be highly efficacious at preventing milder forms of dengue disease, which the currently used case definitions capture (fever + RT-PCR positivity), but they may be highly efficacious at preventing or treating slightly more severe disease, defined here as “moderate.”

We question at what point along the dengue disease spectrum do you begin to identify illness episodes which are clinically relevant and represent individual and public health burden? Is the current “case” definition (fever + PCR positivity) accurately capturing this point? Alternatively, have developers/sponsors and regulatory authorities chosen an endpoint which raises the vaccine efficacy “bar” to focus on prevention of severe and hospitalized cases and does not establish a candidate’s true potential public health benefit by measuring prevention of less severe cases? Because of these questions, we proposed to develop a tool that could measure outcomes of DENV infections to better help elucidate the variable benefits of different vaccine or drug candidates. Importantly, the tool should complement, not replace, the currently used primary efficacy endpoints of severe and hospitalized dengue.

### Dengue Illness Index (DII)

It is important to understand the DII is not designed to categorize dengue disease severity in relation to its pathophysiology or prognosis. The DII is also not designed to replace methods of capturing and characterizing dengue cases currently being employed in interventional trials using World Health Organization 1997 or 2009 guidelines. The DII tool is designed to capture the overall subjective disease experience for an individual based on their identification of having individual symptoms and how the totality of these symptoms impacts the individual’s overall feeling of wellness and ultimately, on daily functional activities, such as those that involve work, school, recreation, and sleep. The DII is meant to complement and supplement current methodologies as stated above.

The DII is intended to capture and quantify the subjective dengue illness experience and supplement the traditional endpoints dengue vaccine and drug developers are using to support their studies. The DII classification defines (1) mild illness as feeling somewhat ill but not enough to effect normal activity and no medication is required for treatment, (2) moderate illness as having some impact on daily activities, and/or non-prescription medication is required to treat signs or symptoms (such as acetaminophen for headache), and (3) severe illness as preventing most or all daily activities, and/or prescription medications are required to treat symptoms, and/or a visit to health care provider is required. Severe illness may or may not result in hospitalization. We assume mild and severe illness experiences will be captured by the standard dengue surveillance systems being utilized in clinical trials. This makes the case for the value of the DII in identifying moderate illness as that which interferes with daily activities short of hospitalization or organ damage.

## Methods

### Workshop to develop standard clinical endpoints to measure moderate and severe dengue in clinical research studies

A scientific working group for the DII was one of three working groups to convene at a workshop attended by 56 participants representing 16 countries, sponsored by the National Institute of Allergy and Infectious Diseases (NIAID) and the Partnership for Dengue Control. This manuscript describes the working group discussions about the DII, its implementation on a diary card referred to as the Dengue Illness Card (DIC), and the results of a Delphi method inquiry that yielded an evolved version of the card proposed for validation and/or piloting. The DII working group was comprised of 10 dengue experts who, along with the members of the other two working groups and non-working group attendees, were identified via referral and selected to achieve a balanced representation of dengue expertise from all sectors and from various global endemic regions. The DII working group members originated from the U.S., Belgium, Brazil, Nicaragua, and Sri Lanka, and represented, alone or in combination, one or more of the following sectors: the government (90%), non-government (10%), academic (60%), pharmaceutical/vaccine development (30%), clinical (40%), and public health (30%).

The first round of comments on the DIC were collected after the first meeting on April 27, 2015, and in preparation for the second meeting on October 29, 2015, which was largely comprised of the same attendees as the first meeting. A number of comments were received from stakeholders in attendance and helped to shape the final product being presented in this manuscript. Some specific comments which lend insight into the varied perspectives include:

### Industry

- Index variables like those proposed to be collected by the DII should be collected prospectively and be a part of the efficacy study design approved before study initiation. A retrospective analysis with a subset of variables that were not pre-specified and were collected from data available in the records will be biased and vary according to the thoroughness of the treating physician.
- Given the time gap between the appearance of the suspected case-defining variable of fever, and virologic confirmation of dengue, index variables would have to be collected on all febrile subjects regardless of diagnosis making data collection and management considerably onerous.
- The DII will need to be adapted for children and infants.
- Collecting variables like those proposed by the DII are not required by regulatory agencies.

### Academia

- Previous prospective field studies have collected data similar to those proposed to be collected by the DII on outpatients, but they did not collect functional impairment endpoints. It should be possible to do this is a prospective way.
- Prospective testing of a tool similar to the proposed DII was completed on a handful of infected, lab-confirmed, travelers from non-endemic country. Based on this experience a composite DII score consisting of the number of symptoms scored daily and over time will be less relevant to the quality of life than the overall functional impairment score.
- Timing may be important with patients who present earlier having higher values for “mean duration of each specific “S&S” but this bias should be balanced in a randomized controlled trial.

The above comments and others were incorporated into modification of the DII; the modified product was then assessed by a Delphi methodology-based query.

### Delphi method

The Delphi methodology-based query was conducted electronically using Mesydel software and commenced with a panel questionnaire that addressed the Overall Approach (OA) of defining and validating the clinical endpoints of moderate and severe dengue in clinical research trials, the overarching topic of the first of the three working groups. Attendees of the first and second meetings, in addition to several non-attendee dengue experts, were invited to participate in the Delphi query and totaled 64 persons, of which 39 accepted an invitation to the initial OA panel. Following this OA panel, participants were given the option of participating in a panel to define the Clinical Endpoints generated from the second working group discussions, or a panel on the third DII working group, or both panels.

A total of 23 panelists self-selected to participate in the DII online Delphi query, of which there were 19 active respondents in round 1, 18 active respondents in round 2, and 10 active respondents in round 3 who commented on the satisfaction of the process. Participants were provided a graphic of the DIC (initially referred to as the Dengue Severity Index (DSI) card) and asked to assess its intuitiveness, visual clarity, practicality, and adequacy. They were further asked if the illness metrics on the card were relevant and sufficient for clinical practice and/or research contexts, and if they would use the DIC. In addition, they were asked specific questions about how and under what conditions the card could be used (e.g., type of study, study population, etc.) and if they could recommend improvements to the card. The majority of questions were open-ended, and participants were given the opportunity to explain or comment further on their answer. Responses in each round were collected over a 2 to 3-week duration.

Responses from round 1 were analyzed and used to modify the card in accordance with participants’ feedback. In round 2, the modified card was presented along with clarifications of its features and intended use, and participants were then asked to assess the quality of the revised card similarly to that done in round 1. The Delphi query concluded with a third round to gauge if respondents felt their comments were taken into consideration, if all relevant issues were addressed, and if they had additional comments.

## Results

### Round 1

In round 1 of the Delphi process, over half of the respondents (53%) felt the card was intuitive, while the other 47% felt it was only relatively intuitive or some combination of not intuitive and not appropriate. When asked to assess the clarity and practicality of the tool, 63% of respondents felt it was clear and practical (or relatively so), while 21% felt it was clear but not practical, and 16% felt it lacked both clarity and practicality. The majority of respondents (79%) felt the card was inadequate and cited reasons that overlapped with those that accounted for the tool’s perceived lack of intuitiveness and clarity/practicality. Specifically, respondents felt the card lacked basic instructions regarding how it should be filled out, how the first day of illness is defined, how symptom severity is rated, and how the index value is calculated. Some respondents questioned the grouping of the signs and symptoms on the card, while others questioned the usefulness of the information captured. There was also concern that card-users would erroneously record the severity of individual symptoms as opposed to the severity of their overall daily illness experience as is intended, and lastly, some respondents felt the card would benefit from the addition of operational guidelines regarding how and when temperature should be measured.

When asked to comment on the sufficiency of the diary card’s metrics for clinical practice and/or research studies, 47% felt it was sufficient, while 37% felt it was insufficient due to a lack of higher grades of severity of the signs and symptoms, as well as inadequate consideration of symptoms such as neurological signs and bleeding. It was also felt that the metrics were not specific enough because the signs and symptoms were not weighted to account for things like frequency of a fever or intensity of headaches. Several respondents commented that the metrics did not aid the assessment of disease progression or were not predictive of disease outcome. One respondent thought that overall illness severity should also be depicted as one’s inability to administer self-care and conduct regular interpersonal relationships, and there were several reiterations that signs and symptoms should not be grouped, but rather, individualized on the card.

Participants were asked in what context the tool would be useful (what phase, for whom and for clinical practice or research). The most cited answer was clinical trials (32%). Other contexts included research (18%); disease burden (14%); before/after medical visit (14%); clinical practice (7%); morbidity (7%); human challenge studies (4%) and surveillance (4%). When asked if they would use the diary card tool, 79% stated they would use it, and when asked for what type of study it should be used, the most frequent answers consisted of vaccine (28%) or clinical trials (23%). Lesser cited studies included clinical studies (9%) and epidemiological studies; fever of unknown origin; dengue research; natural history studies; household contact studies; febrile illness studies and challenge studies, each at 5%. The other 21% of respondents who indicated they would not use the tool stated that it was not specific, clear, practical, intuitive or precise enough and that the card failed to achieve meaningfulness as a stand-alone tool because it would need to be complemented by information collected at a medical visit, or because it did not assess progression towards severe disease.

Suggestions for improvement to the diary card included the addition of more signs and symptoms, as well as simple instructions on how to fill it out and calculate the index value. Some suggested that the format be adjusted to improve ease of use or include a tutorial that informs on the need for urgent medical advice, while other respondents desired more severity detail for the individual signs and symptoms. Several respondents suggested that the card be validated in a prospective study so that grouping of symptoms could be substantiated in an evidence-based manner. One respondent emphasized the need for the tool to be able to predict severe disease outcome, and another respondent suggested eliminating the word “severity” from the name of the card because it tended to be suggestive of the card’s ability to assess the immediate or potential severity of disease as it relates to life-threatening sequelae.

### Round 2

In round 2, a new version of the card renamed “Dengue Illness Card” was presented, which featured formatting suggestions gained from round 1, along with a thorough introduction that emphasized the card’s intended use to better help inform participants’ answer choices in the subsequent round. All 18 of the active respondents felt the card was intuitive, though one participant still felt it was complex and needed to be simplified to better accommodate users with a basic education level. When asked if the card was clear and practical, 83% felt it was, while the 17% who disagreed felt the format and instructions still required improvement such that “first day of illness” is clearly defined, signs and symptoms be adapted for children, and simple, comprehensive instructions be included for the clinical staff administrator and the user. One participant emphasized the need for a strong teacher-student relationship between the clinical administrator and the user to ensure the card is filled out properly, and another respondent desired for the severity scale be simplified.

There was marked improvement in the perceived adequacy of the card as 67% of respondents agreed it was adequate. Reasons cited by the 28% of respondents who did not feel the card was adequate were focused on the calculation of the illness index, stating that severity of illness experience could not be captured in the index calculation without weighting the signs and symptoms.

Almost all the participants (94%) felt the card could achieve its intended purpose. The one participant who disagreed was adamant that the card did not have any utility if it could not guide clinical management or “explore disease severity.” The contexts in which the card was deemed relevant in round 1 were presented in round 2 and respondents were asked to select the top three contexts from the list in which they felt the card is relevant. Eighty-three percent of respondents selected clinical trials, followed by research (72%); disease burden (56%); human challenge studies (44%); before/after medical visit (17%); clinical practice (17%); morbidity (11%) and surveillance (11%). One respondent who felt the tool was appropriate for research and human challenge studies stated that it would be useful for vaccine trials in instances where users were familiar with its format and were in possession of the card before the onset of illness, further expressing concern for the need for febrile individuals to fill the card out retrospectively in clinical practice or clinical trials. Another respondent who felt the card was appropriate for clinical practice and before/after medical visits, commented that the card was not specific enough for clinical trials, research and disease burden.

The large majority of participants (89%) stated they would use the DIC. The resulting list of studies from round 1 for which respondents said the card should be used was presented in round 2 and respondents were asked to select their top three preferred types of studies. The top studies were vaccine trials (68%), clinical trials (68%), and epidemiological studies (37%). Other study types, which some respondents cited were overlapping with one another, included clinical studies (32%); drug trials (32%); natural history studies (32%); challenge studies (21%); dengue research (21%); fever of unknown origin studies (16%); household contact studies (16%) and febrile illness studies (11%). The 11% of participants who stated they would not use the card felt it was not applicable to their clinical-oriented work, did not serve as a prognostic tool to assess disease severity progression, remained non-specific as it was not clear how it was applicable to dengue versus other febrile illnesses, or did not include enough severe endpoints.

When asked if the card was in suitable condition for testing in a real-life setting, 78% felt it was and 22% felt it was not, stating that the format needed to be refined and the instructions needed to be simplified. Additional suggestions for improvements to the card included pilot testing in different populations to account for different ethnic and cultural norms associated with illness, as well as different educational levels.

### Round 3

**Fig 1:**
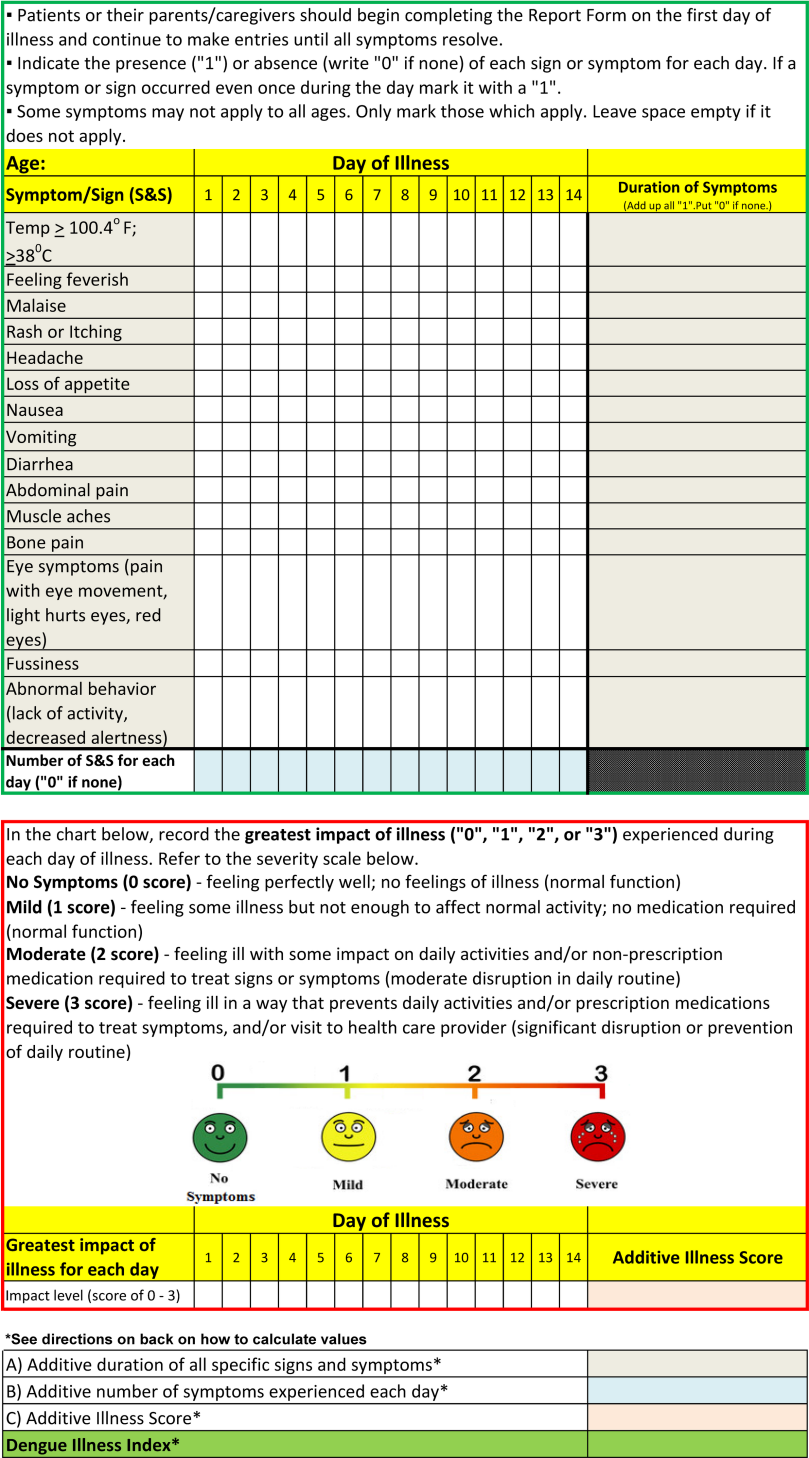
The front side of the final proposed Dengue Illness Card, following suggested modifications from round 1 and 2 of the Delphi. The number of recording days is expanded to 14 days. The scale for the impact of illness is further simplified. The legend for the illness scale better corresponds to the scale, and “greatest level of illness” is changed to “greatest impact of illness”.

**Fig 2:**
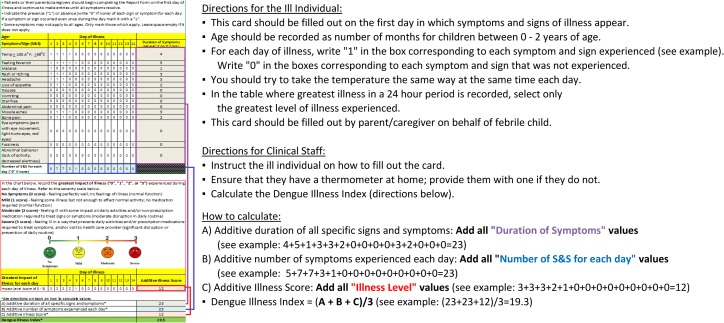
The back of the final proposed Dengue Illness Card, following suggested modifications from round 1 and 2 of the Delphi. Detailed instructions for the ill individuals as to how to measure temperature and fill out the card; instructions for clinical staff, as well as an example diagram of the card with instructions on how to calculate the index.

In round 3, participants were presented with the results of round 2 along with a graphic image of the final DIC (Fig 1 and Fig 2). When asked about their overall satisfaction with the DIC Delphi method, >88% felt their input was taken into consideration, felt they received sufficient feedback throughout the Delphi method, and felt that all relevant issues were addressed. Questions addressing the demographic profile of the respondents revealed that they self-identified with the following sectors: academic (44%); clinical (30%); government (30%); pharmaceutical/vaccine development (30%); public health (26%) and non-government (9%). Their work was relevant to the United States (44%); Central and South America (44%); Southeast Asia (22%); Europe (13%) or globally (13%). The majority of respondents originated from the United States (57%), while others originated from Europe (13%); Southeast Asia (13%) and Central and South America (9%). All but one respondent worked in a dengue-relevant capacity.

## Discussion

Dengue is a continually expanding global health problem causing significant human suffering and utilization of scarce health care resources in numerous developing countries. As the environment becomes more hospitable to expanding *Aedes* breeding habitats and the DENVs are disbursed rapidly in viremic travelers there is bound to be a worsening of the global dengue pandemic. Safe and highly effective countermeasures are required to protect against dengue disease and to reduce the associated public health burden. Developing such measures has been a significant R&D challenge.

One development challenge, in our opinion, has been selecting the appropriate definition for what constitutes a “dengue case.” Counting such cases in vaccine recipients versus control or placebo recipients determines the intervention’s efficacy. The definition needs to meet regulatory rigor, be objectively measured, and be generalizable across different countries and health care cultures and practices. The current definition of a case, i.e., one to three days of fever and PCR or NS1 antigen positivity, does not seem to lend itself to the goal of developing vaccines and drugs to protect people against clinically relevant dengue. Relevant dengue is the disease that matters from a human suffering, morbidity, and mortality perspective as well as what drives health care seeking behavior and lost productivity. One to three days of illness may not rise to the point of being clinically relevant using these criteria, and PCR or NS1 positivity may not be accompanied by symptoms; this is what occurs in the majority of DENV infections, which are asymptomatic. For this reason, some developers also desire to measure efficacy against severe or hospitalized disease, two disease categories also preferred by regulatory authorities.

Because severe dengue occurs in a minority of infections (2%-4%), it is not reasonable to have severe dengue, as defined by WHO guidelines or product-specific criteria defined by Data Safety Monitoring Boards or their equivalent, as primary outcomes in a trial. The sample size required to capture enough severe dengue to draw conclusions on countermeasure performance with sufficient power would be immense. Although one developer did report efficacy against hospitalized and severe disease the overall numbers are low, confidence intervals wide, and the potential for major shifts in the data, and outcome, with a few more cases assigned to one category or another is very real. Another issue is the relative burden of inpatient versus outpatient dengue in terms of financial cost and disability-adjusted life years lost. Outpatient dengue represents a significant economic cost and disease burden.[3, 20–22] The authors propose that these two extreme measures miss an entire spectrum of dengue disease which is clinically relevant and represent a sizable personal and societal burden. For this reason, we sought to develop a tool which could capture the personal subjective experience of dengue illness and assign it a numeric value to be used in the context of dengue or dengue countermeasure research. The numeric value will allow for contrast and comparison between interventions and different populations, measured once or repeatedly over time.

We learned much during the Index development process. First, there was general agreement that dengue researchers may be missing a large swath of dengue which is clinically relevant but not captured with most study designs focused on fever + PCR positivity or severe dengue. Second, we observed a renewal of the debate surrounding use of the term, “severe,” and the confusion it created when it was liberally applied to a subjective personal experience (i.e. volunteer documented his headache as severe), refer to a guideline (i.e. WHO 1997, 2009, IDMC derived, etc.), or describing the pathophysiology of dengue disease (i.e. platelet count, rise in hematocrit, bleeding, plasma leakage, etc.). For this reason, we adjusted our tool’s name from a Dengue Severity Index to a Dengue Illness Index. Third, the chasm between the clinician and the researcher remains intact. The needs of a busy clinician are different from the needs of a clinical researcher executing a field trial of a drug or vaccine candidate, and these differences were observed while we assessed the tool and its potential applicability. Finally, there was general agreement that a tool such as the one we propose could generate useful data. The tool could add a valuable and alternative perspective to the assessment of drug or vaccine candidates in field trials, human challenge studies, and epidemiological studies. However, deployment and use of the tool during such studies would be required for “validation”, that is, proving the DIC can be used easily and that the DII can generate clinical data of good quality.

### Next steps

The Dengue Illness Card will advance in two parallel paths. The first path will be immediate application in its current form to ongoing field investigations or investigations where execution is imminent. We communicated with the researchers and their teams that the tool would be made available for their use. The tool was designed to not be cumbersome and to fit into, not replace, the current processes researchers use to collect data during their studies. Once the tool is utilized, data collected, and analysis complete the research teams can decide if it added value to their research. If it did, they could move forward with the tool. If it did not, they could reject further use; either outcome would be informative to the tool developers.

The second path would be to allow researchers to take the tool and manipulate it to meet their specific objectives and needs. For example, a pharmaceutical company developing a dengue drug or vaccine may want to use the tool as a foundation to develop a more advanced tool which captures more data or could withstand a more rigorous regulatory assessment. The tool could also be simplified. For example, the 24-hour functional level of illness score (Fig 1) can be calculated and used as stand-alone value to compare the functional burden of an illness episode in trials of different vaccines or drugs. Once reworked, the tool could be prospectively deployed into vaccine or drug trials, used alongside the standard methods of data capture, and its usefulness evaluated and “validated.”

In conclusion, we contend there is merit to developing and deploying a data capture tool which aims to identify the spectrum of dengue disease which is relevant by many metrics but not always captured using the current and broadly applied data capture methods. It is our hope through meetings sponsored by government, industry and foundations and through this publication, researchers will be willing to include this tool into their research plans and to explore the tool’s performance and value, or lack thereof, in investigations of countermeasures to reduce dengue’s global burden.
